# Rice Grain Size and Quality

**DOI:** 10.1186/s12284-022-00579-z

**Published:** 2022-07-01

**Authors:** Kiyosumi Hori, Jian Sun

**Affiliations:** 1 Institute of Crop Science, National Agricultural and Food Research Organization, Tsukuba, 305-8518 Japan; 2 Shenyang Agricultural University, Rice Research Institute, Shenyang, 110866 China

Rice (*Oryza sativa* L.) is one of the most important cereal crops in the world and is a dietary staple food for over half of the world’s population. An increase in grain yield is continuously required to ensure food security. In addition to yield, grain quality is also paid particular attention by rice consumers, food industries, farmers, and seed producers (Champagne et al. 1999; Fitzgerald et al. 2009). Grain size and quality essentially determining the market price of rice. The global improvement in living standards is enhancing consumer preference for high-quality grains in rice cultivars. Therefore, grain quality, including grain size, is a major target in rice breeding programs globally (Bao 2014).

Grain quality is a complex quantitative trait because it is determined by many characteristics, including physical appearance, milling quality, nutritional value (grain components), aroma, and cooking and eating quality (Fig. 1). It is known that a lot of genes are involved in controlling each grain-quality trait. Decoding whole-genome sequences of rice cultivars has accelerated the detection of genetic factors (QTLs) and isolation of genes for the grain-quality traits. For example, grain size shows high heritability, and studies conducted so far have isolated several responsible genes, such as *GS3*, *GW2*, *GS5*, and *TGW6* (Li et al. 2018). In addition to these major genes, a lot of minor QTLs are associated with grain size (Nagata et al. 2015). Chalkiness grains have opaque spots in endosperm and reduce grain quality because of decreasing milled rice ratio, especially *indica* rice cultivars of slender grains. The high temperature at grain-filling stage increases chalkiness and decreases grain quality in *japonica* rice cultivars of shorter and wider grains. Therefore, there have been a lot of genetic studies to detect QTLs and genes for grain chalkiness, such as *Chalk5*, *Flo2*, and *GIF2* (Li et al. 2014). Grain components largely contribute to eating and cooking characteristics. Starch is the main component of more than 95% of dry weight in rice grains. Rice starch is divided into amylose with linear connected glucose and amylopectin with branched glucose chains. The *GBSSI* (*Wx*) gene has the main function for amylose biosynthesis in rice grains, determines glutinous and non-glutinous cultivar types, and primarily affects eating and cooking qualities. The *SS2a* (*Alk*) gene has a function for elongating branched glucose chains of amylopectin in rice endosperms. Allelic variations of the *SS2a* gene change the viscosity and gelatinization temperature of rice starch. Although the *GBSSI* and *SS2a* genes determine major genetic effects for natural variations of starch properties, several QTLs for controlling amylose content, amylopectin chain length distributions, and eating and cooking qualities are detected on all of the rice chromosomes (Hori et al. 2021). Protein (amino acids), lipids, and elements are essential for enhancing human nutrition and also affecting differences in eating and cooking qualities.

**Fig. 1:**
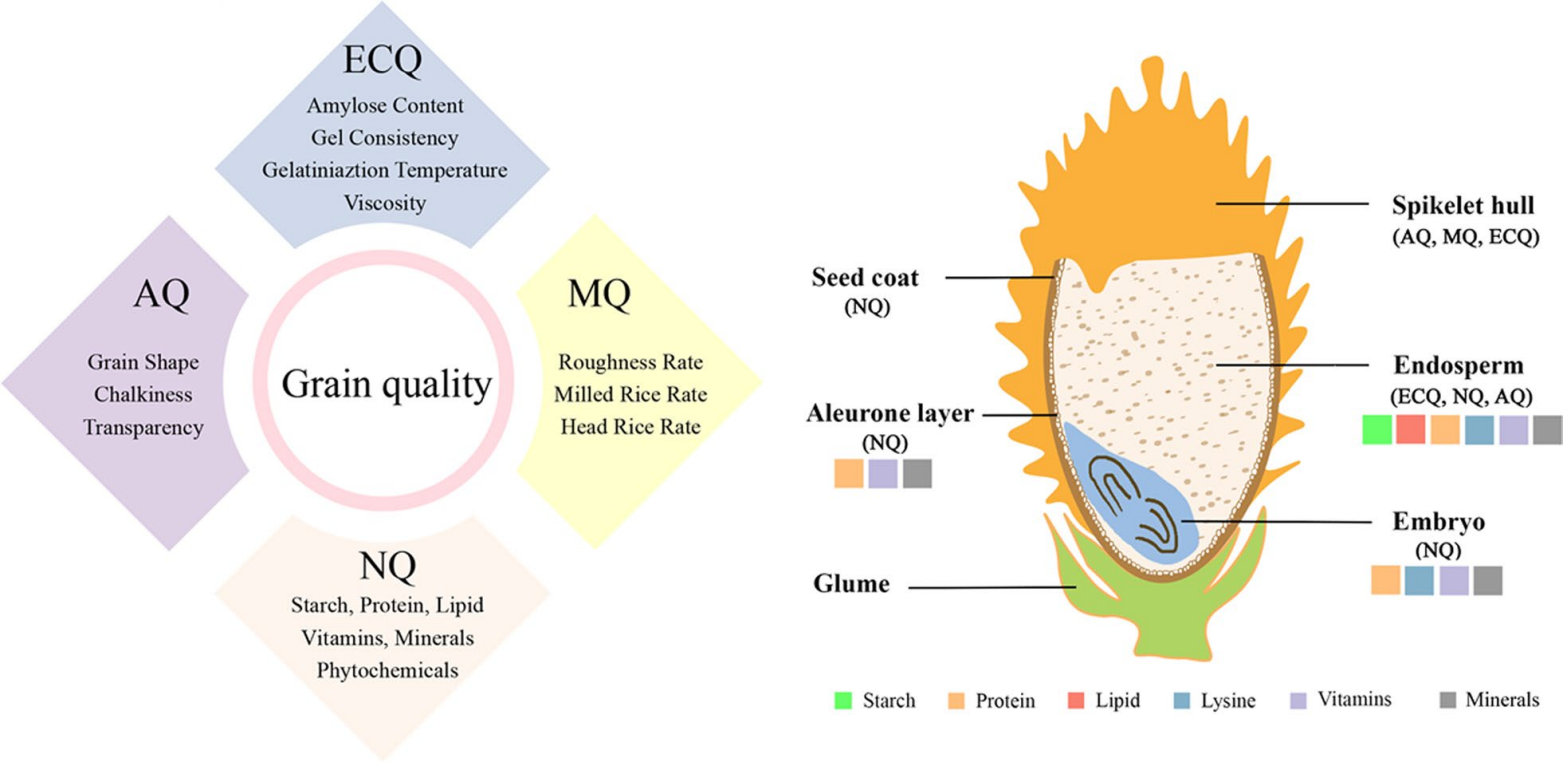
This special issue covers the latest genetic and molecular insights on rice grain size and quality. The images are cited from Li et al. (2022a, Rice 15:18) showing the longitudinal section of a mature rice grain (right) and the four components that comprise the scope of rice quality (left)

In this special issue, we collect review and original research articles covering the recent significant advances in identifying grain-quality genes, elucidating their gene regulatory network, and applying fundamental research efforts to breeding programs. Since the first rice genome sequence of ‘Nipponbare’ was released in 2004 (IRGSP 2005), a lot of molecular genetic studies have been performed on rice by using natural sequence variations including wild relatives, mutant populations, transgenic lines, genome editing lines, and omics technologies, such as genomics, transcriptomics, proteomics and metabolomics, and epigenetic analyses. By using rice genome sequences and map-based cloning procedures, more than 137 genes have been isolated up-to-now and their molecular functions for controlling the complex grain-quality traits such as grain shape (appearance), milling quality, nutritional quality, and eating and cooking qualities have been elucidated (Li et al. 2022a). Moreover, these studies clearly revealed major regulatory mechanisms of the isolated genes for grain size, such as grain length, grain width, and their ratio, and grain components, such as starch, protein, lipids and elements. Nutrition uptake and transport are essential for both plant growth and grain ripening. Zhang and (2022) summarized so far isolated genes for uptakes, transport, re-translocation, and volatilization of selenium as an example. Recent genetic studies are also tackling a number of novel QTLs and genes for controlling grain-quality traits (Li et al. 2022b). Novel grain length QTL of *qGL11* was fine mapped within the 810-kbp interval region on chromosome 11. Gene x gene interaction (combination of gene alleles) creates novel grain quality that does not exist in natural variation of rice cultivars. Combinations of deficient alleles among starch biosynthesis genes *GBSSI*, *SS2a*, *SS3a*, *SS4b*, *ISA1*, and *BE2b* showed unique starch properties such as increasing resistant starch for improving human health benefits (Fujita et al. 2022; Miura et al. 2022). Information on genetic diversity and gene allelic variations are useful for applications of fundamental genetic studies to develop novel breeding lines. Near-isogenic lines (NILs) are good candidates for breeding new rice cultivars and for introducing specific gene alleles for improving grain quality and increasing grain yield. The deficient allele of *PDIL1-1* gene increases grain chalkiness but improves rice flour qualities such as small particle size of rice flour and large loaf volume of rice bread. NILs introducing the deficient allele of *PDIL1-1* gene showed phenotypic alterations between different genetic backgrounds of two rice cultivars (Hori et al. 2022). The deficient allele of the *Dep1* gene makes dense and erect panicles in rice plants, and increases grain yield and nitrogen use efficiency. Chen et al. (2022) revealed that NILs introducing the erect type allele of the *Dep1* gene showed additional positive effects on protein accumulation but adverse effects on eating quality. The recent rapid development of genome editing technology can easily create knockout and modification of target gene alleles. Several genome editing studies successfully improved the number of specific grain components such as amylose, amino acids, fatty acids, β-carotene, 2-Acetyl-1-pyrroline (for fragrance), and cadmium (elements) by enhancing biosynthetic pathways or suppressing catabolic pathways (Sukegawa et al. 2022). Genome editing technology would be a powerful tool for the development of novel rice cultivars.

Based on the critical scientific information conveyed in these articles, we realized that understanding the genetic basis is the prerequisite to improve grain-quality traits in rice. We hope that the topics in this special issue will enhance the efficiency of breeding selections for improving grain quality with other agronomic traits, including high grain yield and strong disease/insect resistance.

## Data Availability

All datasets are included in the article.
